# Transdiagnostic phenotypes of compulsive behavior and associations with psychological, cognitive, and neurobiological affective processing

**DOI:** 10.1038/s41398-021-01773-1

**Published:** 2022-01-10

**Authors:** Lauren Den Ouden, Chao Suo, Lucy Albertella, Lisa-Marie Greenwood, Rico S. C. Lee, Leonardo F. Fontenelle, Linden Parkes, Jeggan Tiego, Samuel R. Chamberlain, Karyn Richardson, Rebecca Segrave, Murat Yücel

**Affiliations:** 1 BrainPark, The Turner Institute for Brain and Mental Health, School of Psychological Sciences and Monash Biomedical Imaging Facility, Monash University, Clayton, Australia; 2 Research School of Psychology, ANU College of Health and Medicine, The Australian National University, Canberra, Australia; 3 D’Or Institute for Research and Education and Anxiety, Obsessive, Compulsive Research Program, Institute of Psychiatry, Federal University of Rio de Janeiro, Rio de Janeiro, Brazil; 4 Department of Bioengineering, School of Engineering & Applied Science, University of Pennsylvania, Philadelphia, PA 19104 USA; 5 Neural Systems and Behaviour Lab, The Turner Institute for Brain and Mental Health, School of Psychological Sciences and Monash Biomedical Imaging Facility, Monash University, Clayton, Australia; 6 Department of Psychiatry, University of Southampton, Southampton, UK; 7 Southern Health NHS Foundation Trust, Southampton, UK

**Keywords:** Prognostic markers, Biomarkers

## Abstract

Compulsivity is a poorly understood transdiagnostic construct thought to underlie multiple disorders, including obsessive-compulsive disorder, addictions, and binge eating. Our current understanding of the causes of compulsive behavior remains primarily based on investigations into specific diagnostic categories or findings relying on one or two laboratory measures to explain complex phenotypic variance. This proof-of-concept study drew on a heterogeneous sample of community-based individuals (*N* = 45; 18–45 years; 25 female) exhibiting compulsive behavioral patterns in alcohol use, eating, cleaning, checking, or symmetry. Data-driven statistical modeling of multidimensional markers was utilized to identify homogeneous subtypes that were independent of traditional clinical phenomenology. Markers were based on well-defined measures of affective processing and included psychological assessment of compulsivity, behavioral avoidance, and stress, neurocognitive assessment of reward vs. punishment learning, and biological assessment of the cortisol awakening response. The neurobiological validity of the subtypes was assessed using functional magnetic resonance imaging. Statistical modeling identified three stable, distinct subtypes of compulsivity and affective processing, which we labeled “Compulsive Non-Avoidant”, “Compulsive Reactive” and “Compulsive Stressed”. They differed meaningfully on validation measures of mood, intolerance of uncertainty, and urgency. Most importantly, subtypes captured neurobiological variance on amygdala-based resting-state functional connectivity, suggesting they were valid representations of underlying neurobiology and highlighting the relevance of emotion-related brain networks in compulsive behavior. Although independent larger samples are needed to confirm the stability of subtypes, these data offer an integrated understanding of how different systems may interact in compulsive behavior and provide new considerations for guiding tailored intervention decisions.

## Introduction

Traditional classification systems, such as the Diagnostic and Statistical Manual (DSM) and International Classification of Diseases (ICD), remain the primary means for classifying psychopathology. However, there is mounting evidence that diagnostic categories do not capture the natural organization of psychopathology symptoms, thus impeding identification of underlying neurobiological substrates [1–5]. This has led to calls for empirically-based approaches to study psychiatric nosology that will foster the neuroscientific discovery of pathogenic mechanisms across multiple levels of analysis [i.e., symptom, cognitive, neurobiological, [6–9]]. Data-driven approaches are essential in identifying psychiatric biomarkers [10, 11] and the development of more effective, personalized treatments [12, 13].

Data-driven clustering, a machine learning approach that learns patterns from data in the absence of group labels (e.g., disorder groups), is a promising method for reclassifying mental disorders. In psychiatry, clustering has commonly been applied to neurobiological data [5, 14–17]. While such brain-based clusters may have the potential to unearth biological substrates of psychopathology [5, 15, 17], the variability associated with biological data risks detection of biotypes unrelated to psychiatric presentation [18]. An alternative approach is to apply clustering to so-called intermediate phenotypes [1, 2, 4, 19–21]. Here, intermediate phenotypes are derived from the behavior and cognitive function rather than just clinical symptomatology. Critically, previous work has shown that intermediate phenotypes track variation in clinical symptoms across multiple disorders [3], and can be mapped onto underlying brain structure and function [2, 19, 21]. This approach has been shown to be more sensitive to detect neural correlates in psychiatric patients than conventional case-control comparisons [2, 19], revealing new insights into psychopathology.

Compulsivity is an intermediate phenotype, defined by rigid, repetitive, and functionally impairing behaviors [22], that is relevant to understanding and treating a variety of mental health disorders [23]. Individual differences in compulsivity underlie vulnerability to disorders including obsessive-compulsive disorder (OCD), substance and behavioral addictions [2, 24–26]. Compulsivity also exists outside psychiatric diagnoses, with problematic behavior frequently evident at subclinical and community-based levels [3, 4]. Despite shared cognitive and neurobiological underpinnings [24, 25], the causes of compulsive behaviors have traditionally been examined in the context of specific diagnostic categories [27–29] or rely on one or two laboratory measures to explain phenotypic variance [30–32]. This is problematic as compulsive behavior is not constrained to one clinical category and a single outcome measure can rarely be pathognomonic for complex psychiatric behavior, with disruptions often expressed across several measures.

Our recent work has begun to address these issues, identifying compulsivity as a transdiagnostic phenotype, measurable dimensionally in both the general population and traditional diagnostic categories [1, 4]. We have shown that it is closely tied to cortical-striatal-thalamic-cortical function [2]. That is, individual differences in effective connectivity across conditions such as OCD and gambling disorder are better characterized by transdiagnostic measures of compulsivity rather than comparisons based on diagnostic labels. This demonstrates that compulsivity has the potential to explain individual variance at both the symptom and neurobiological levels. However, compulsivity is highly multifaceted [33, 34] and our understanding of how it should be operationalized and measured remains in its infancy.

In particular, compulsivity research has tended to focus on ‘cool’ cognitive processes (i.e., processes that operate in affectively neutral contexts [35]) over ‘hot’ processes (i.e., processes that operate in motivationally and emotionally significant situations). This is despite the widely accepted role of affect dysregulation in addiction and OCD [24, 36] and research showing disturbances in affective processes may contribute to symptom presentation [25, 28, 34]. For example, biased learning of emotionally-relevant stimuli and responses may promote persistence of maladaptive behavior in OCD [37, 38] and addiction [38, 39]. Here, we have conceptualized poor affect processing and regulation as being implicated in compulsive behavior and therefore selected a set of measures tightly linked to intermediate affective processes relevant to compulsivity. Intermediate affective processes being those that mediate the relationship between affective neurocircuitry (e.g., limbic circuits) and overt mood symptoms (e.g., anxiety).

Firstly, the Cortisol Awakening Response (CAR) is the increase in cortisol concentration within the first hour of awakening and is an indicator of hypothalamic-pituitary-adrenocortical (HPA) stress-system function [40]. Stress and hormonal stress response systems have been shown to promote habitual behavior in compulsive disorders, particularly in addiction [41–43]. Second, biases in valence-based attentional deployment underpin emotional problems in a number of mood-related clinical conditions (e.g. anxiety, depression; [44]) and are observed in substance use [38, 45], problem gambling [46], and binge eating [47, 48]. Therefore, a reward versus punishment learning paradigm was used to assess attentional biases toward positive and negative stimuli [49]. Finally, psychological self-report measures of stress, experiential avoidance, and compulsive behavior respectively, assessed poor perceived coping with emotional situations, disproportionate negative evaluation of aversive emotions, and over-use of avoidance behaviors to manage emotions.

Evidence from animal and human studies indicates a crucial role of the amygdala in affective processing [50]. Interactions among large-scale brain networks and the amygdala subserve many of the psychological and cognitive processes involved in affective processing [51–53]. This was illustrated in a study showing risk tolerance to be most strongly related to amygdala-based resting-state node strength when compared to all other brain nodes [54]. Moreover, resting-state functional connectivity (rs-FC) between the amygdala and medial prefrontal cortex (mPFC), a region within the emotional-appraisal network [55], made one of the greatest contributions in predicting risk tolerance. Higher rs-FC of the amygdala with mPFC (and other cortical regions) are thought to reflect the capacity for greater top-down modulation [56–58], relating to less affective reactivity and compulsivity.

In this proof-of-concept study, our broad aim was to identify naturally occurring transdiagnostic phenotypes of compulsivity, whilst including measures of affective processing that have so far received little attention. To do this, we first applied data-driven clustering to detect “hidden” subtypes based on different combinations of compulsivity and affective processing, within a sample of individuals exhibiting compulsive behavioral patterns in alcohol use, eating, cleaning, checking, or symmetry. We utilized multidimensional indicators to capture affective compulsivity across psychological, cognitive, and biological levels of function. Due to the novelty of the current analysis, we included additional measures of overt clinical symptoms to describe additional sample characteristics, to support the interpretation of the data, and aid replicability across community samples in future studies. Finally, to determine if subtypes reflected underlying neurobiological differences, we investigated whether they mapped onto distinct patterns of amygdala-based rs-FC.

Based on the nature of phenotypes that have emerged in other transdiagnostic, multidimensional clustering studies recruited from the general community [19, 20], we anticipated obtaining a final solution containing at least three subtypes. Namely, (1) low risk and relatively normal expression across measures of compulsivity and affective processing, (2) intermediate with evidence of mild or more localized disruptions across measures, and (3) poor outcomes across multiple measures. Subtypes were expected to exhibit outcomes consistent with these profiles on validators. Finally, we anticipated subtypes characterized by disruptions on compulsivity and affective processing measures to exhibit reductions in amygdala-based rs-FC.

## Materials and methods

### Participants

Forty-five participants (25 female; aged 18–46 years) reporting current and persistent engagement in either an OCD- or addiction-related compulsive behavior were recruited from the community (detailed further in Supplementary Material). Participants provided informed consent as part of a larger behavioral intervention trial targeting mild to moderate compulsive behaviors. Data used in the current study is from the baseline assessment, prior to any intervention. Compulsive behavior was defined as a score ≥ 5 on the compulsive subscale of the self-report Yale-Brown Obsessive-Compulsive Scale (Y-BOCS; modified for alcohol and eating) over the past 3-months. A subscale score of ≥ 5 is indicative of mild OCD [59] without necessarily meeting diagnostic threshold for the disorder. Participants were excluded for lifetime and current psychological, neurological, and medical conditions that could affect testing procedures (full inclusion and exclusion criteria detailed in Supplementary Material). All experiments were performed in accordance with relevant guidelines and regulations of Monash University Human Research Ethics (Project ID: 0437).

### Materials

Additional detail on the materials, MRI data acquisition and pre-processing can be found in Supplementary Material.

#### Compulsive behavior

Originally developed for OCD, the Y-BOCS has been adapted to measure addiction-related compulsive behaviors [60, 61]. Adapted versions used in this study measure self-reported obsessions and compulsions over the past 3-months related to either checking, achieving symmetry, cleaning, alcohol consumption, or eating. Where participants endorsed multiple behaviors, the Y-BOCS with the highest score was used in the analysis. While the inclusion criteria of ≥ 5 on the compulsive subscale of the Y-BOCS was used to ensure the data captured self-reported compulsive phenotypes associated with the repetitively performed behaviors, the total score (compulsions and obsessions subscales) was used in the final cluster analysis. The total score integrates complex composite features (*thoughts* and *behaviors*) of compulsivity [62–65] in order to investigate the natural organization of associated psychological, cognitive, and neurobiological processes. Moreover, obsessions and compulsions tend to cluster together and there is often limited utility in differentiating them [66, 67]. Y-BOCS total scores can be interpreted as subclinical (0–7), mild (8–15), moderate (16–23), severe (24–31), and extreme (32–40).

#### Behavioral avoidance

The tendency to use behaviors to reduce or avoid negative mood states was assessed using the behavioral avoidance subscale of the Multidimensional Experiential Avoidance Questionnaire 62-item (MEAQ-62) [68]. This subscale measures overt avoidance of distressing or uncomfortable situations, whereby higher scores index increased use of behavioral strategies to avoid negative internal experiences. Normative data shows community-based adults score *M* = 34.40, *SD* = 10.41, while psychiatric patients score *M* = 42.36, *SD* = 11.13.

#### Stress

The Perceived Stress Scale (PSS) [69] assessed the degree to which participants felt they could cope and respond to stressors. Higher scores reflect increased distress while lower scores reflect good coping or fewer stressors/challenges present. Normative data from community-based adults aged 18–29 years elicited *M* = 14.2, *SD* = 6.2.

#### Valence learning bias

A computerized assessment called “BeanFest” served as our neurocognitive measure of reward vs. punishment learning biases [49]. The task measures individual differences in learning based on wins and losses. Participants attempt to win points and avoid losses by learning which beans are rewarding (win) and punishing (loss). After the learning phase, participants classify beans as “helpful” or “harmful” to assess learning of rewarding vs punishing beans (i.e., valence learning bias). Valence learning bias is calculated as the difference between the proportion of rewarding and punishing beans classified correctly. Scores can range from −1.00 to 1.00, whereby scores below zero indicate punishment learning bias and scores above zero indicate reward learning bias.

#### Cortisol awakening response

Participants collected three saliva samples per day over two consecutive working days (awakening (t_0_), 30-min (t_30_), and 45-min after awakening (t_45_)). To quantify the cortisol awakening response (CAR), the CAR salience index (difference between mean secretion rate before and after 30-min: Formulaic expression: ((t_30_ − t_0_)/30) − ((t_45_ − t_30_)/15)) was used as it has recently been shown to perform significantly better than traditional CAR calculations at revealing trait-like individual differences [70].

### MRI data acquisition and pre-processing

#### Acquisition

The dataset was acquired on a Siemens MAGNETOM Skyra 3 T scanner. T1-weighted (T1w) images are TE = 2.55 ms, TR = 1.52 s, flip angle = 9°, 208 slices with 1 mm isotropic voxels. EPI images for resting-state fMRI (rs-fMRI) are TE = 30 ms, TR = 2.5 s, flip angle = 90°, 189 volumes, 44 slices. Total resting-state scan time = 7.88 min. Participants were asked to look at a fixation cross on the screen and not fall asleep.

#### Pre-processing

T1w and rs-fMRI images were pre-processed using fmriprep (version 1.1.1) on a CENTOS 7 cluster computing system (www.massive.org.au), including: distortion correction, head motion correction, slice timing, special normalization to standard space (i.e., Montreal Neurological Institute [MNI] space), confound signals removal using ICA-AROMA and CompCor and smoothing with 6 mm Gaussian kernal. The rs-fMRI images were de-trended and band-pass filtered at 0.01–0.1 Hz. The rs-fMRI images were used as input to calculate the amygdala-based functional connectivity network. Bilateral Amygdala seeds were generated from Harvard-Oxford subcortical template using FSL. The probability template is the threshold at 90% and saved as the seed of a binary mask. Functional connectivity (FC) maps were generated using RESTplus V1.22 [71]. Further voxel-based statistical analysis on FC maps is detailed in the “Statistical analyses” section.

### Procedure

With the exception of saliva samples, all data collection was conducted at Monash University BrainPark, Melbourne. Participants completed two 90-min research sessions which were conducted within 1 week of each other. Session one involved consent, diagnostic interview, and questionnaires. Session two comprised the MRI brain scan and cognitive assessment. Saliva sampling protocol was completed at the participants’ homes using a home testing kit (SalivaBio) within 1 week of completing session two. See Supplementary Materials for detail on saliva collection, storage, and analysis.

### Statistical analyses

#### Identifying clusters

We clustered individuals using measures of compulsivity (total Y-BOCS), behavioral avoidance (MEAQ), stress (PSS), valence learning bias (BeanFest), and CAR (MnInc). Each variable was *Z*-scored so that it contributed equally to the distance measure. A combination of hierarchical and k-means cluster analyses (performed in IMB SPSS Statistics 25) was used to detect distinct subtypes. A hierarchical agglomerative method (Ward’s method) with squared Euclidean distance was first implemented to explore the number of clusters for entry into the *k*-means analysis. The number of clusters was decided following examination of the dendrogram, and by identifying large differences between consecutive numbers in the agglomeration schedule [72]. In most instances, a two-cluster solution would be chosen at this stage because of the natural increase in heterogeneity that comes from the reduction in clusters [72]. We restricted the solution to three clusters or more based on (1) findings from similar multidimensional clustering studies which have shown at least three subtypes typically exist [19, 20] and (2) the limited potential for a two-cluster solution to elicit meaningful profiles across multiple dimensions of function.

The stability of the final solution was confirmed through several assessments. First, the agreement between the two method solutions (i.e., Ward’s method and *k*-means) was assessed using Cramer’s V test. Next, the final solution (derived from the *k*-means analysis) was further assessed by running ten passes with different random seed starting points [73] and comparing results by Cohen’s kappa (*k*) and intraclass correlation coefficient (ICC). Overall, a *k* < 0.2 reflected poor agreement; 0.21–0.4, fair; 0.4–0.6, moderate; 0.61–0.8, good; and *k* > 0.81, very good. Finally, stability of the cluster solution was tested using a bootstrap technique. Using the R package “fpc” version 2.1.9, the Jaccard coefficient was calculated to compute the structural similarity (ranging from 0 to 1) of 2000 resampled clusters with those derived from the original data [74]. Valid, stable clusters should yield Jaccard coefficients ≥ 0.75 and values above 0.85 are considered “highly stable”. Discriminant function analysis (DFA) was run with cluster input variables as predictors and cluster membership as criterion variables to examine the cluster solutions’ classification accuracies and inspect the separation of the clusters in discriminant function space.

#### Internal validation measures

We selected measures of clinical characteristics with which to internally validate the cluster solutions. These included measures of anxiety (State-Trait Anxiety Inventory Y2), depression (Centre for Epidemiologic Studies Depression Scale Revised), and intermediate process related to compulsivity (intolerance of uncertainty [IUC; Intolerance of Uncertainty Scale] and impulsive urgency [UPPS-P Impulsive Behavior Scale]). MANOVAs, ANCOVAs, and chi-squared analyses were used where appropriate (two-sided), with Bonferroni adjustment for multiple comparisons on post hoc analyses. Amygdala-based FC maps for each subtype were generated using one-sample *t-*test to visually compare the network pattern (SPM12 software). An F-contrast was used to examine the subgroup effect on the amygdala-based rs-fMRI network, controlling for age and sex. Then, independent t-tests were conducted to examine directional differences between each subtype. For each comparison, results were first thresholded at *p*_uncorrected_ < 0.001 with cluster size > 10, then corrected for multiple comparisons error at the cluster level of *p* < 0.05, using family-wise error (FWE) correction. Further detail on statistical analysis in Supplementary Methods.

## Results

### Sample size

There is no generally accepted minimum sample size in clustering, however a sample size of at least 2^*m*^, where *m* equals the number of clustering variables has been recommended [75]. The minimum sample size for the current investigation is 2^5^ = 32.

### Descriptive analyses

Primary compulsions, identified by the Y-BOCS compulsive subscale, included checking (*n* = 5), achieving symmetry (*n* = 13), cleaning (*n* = 9), alcohol consumption (*n* = 6) or eating (*n* = 12). Twenty two participants met diagnostic criteria (using the MINI International Neuropsychiatric Interview for DSM-5) for current OCD (*n* = 12), binge-eating disorder (*n* = 4) and alcohol-use disorder (*n* = 6). Variable means and standard deviations, missing data, outliers and assessments of normality, and multicollinearity are detailed in Supplementary Results and Table S1. Pearson’s correlations between variables ranged from 0.02 to 0.52 (Table S2).

### Hierarchical cluster analysis

Cluster analysis based on Ward’s method provided the greatest support for two- and three-cluster solutions. The dendrogram supported up to four potentially occurring clusters (Supplementary Fig. S1). However, the percentage change in the agglomeration coefficient argued against a four-cluster solution, as the increase exceeded that of the previous stage [Supplementary Tables S3 and S4; [72]]. The largest change was seen in the two-cluster solution (35.50%), followed by the three-cluster solution (20.58%). Given we restricted our solution to three or more clusters, the three-cluster solution was carried into further analyses.

### *K*-means cluster analysis

K-mean cluster analysis was next implemented, specifying a three-cluster solution. There was excellent agreement between Ward’s method and K-means clustering, with Cramer’s V = 0.86 and Cohen’s kappa = 0.83, both *p* < 0.001. The three-cluster solution showed excellent stability when the seed starting point was randomly altered ten times. There was high profile similarity (ICC > 0.90) between all solutions and they all demonstrated very good to excellent agreement with the original solution (*k* = 0.70–1.00). Average Jaccard bootstrap values for clusters were 0.77, 0.80, and 0.96, indicating the clusters were valid and stable. DFA indicated the three subtypes were adequately separated in discriminant function space and that 100% of cases were correctly classified. See Supplementary Fig. S2 for visualization of clusters in two-dimensional space.

### Subtype characteristics

**Fig. 1:**
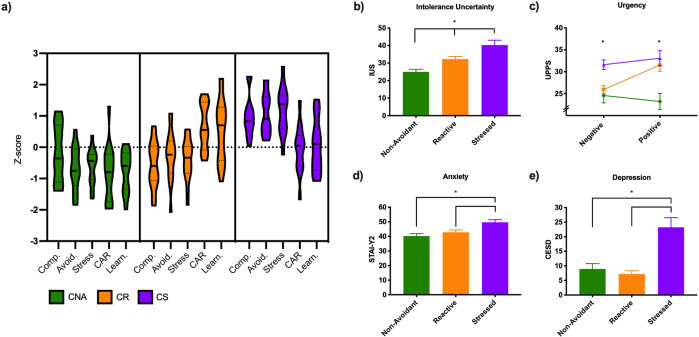
Subtype characteristics on cluster variables and internal validation measures. **a** Violin plots for each of the cluster variables by subtype and comparison of subtype differences on **b** intolerance of uncertainty **c** positive and negative **d** anxiety and **e** depression. CNA (green) Compulsive Non-Avoidant; CR (orange) Compulsive Reactive; CS (purple) Compulsive Stressed subtype. Comp. = Y-BOCS z-score for primary compulsion; Avoid. = MEAQ behavioral avoidance *z*-score; Stress = PSS z-score; CAR = cortisol awakening response salience z-score; Learn. = valence learning bias z-score as measured by the BeanFest task. IUS Intolerance of Uncertainty Scale, UPPS = UPPS-P impulsive behavior scale. STAI-Y2 State-Trait Anxiety Inventory Y2 (trait); CESD Centre for Epidemiologic Studies Depression Scale Revised. Bars represent group means and error bars represent standard error. *p < 0.05.

Subtype profiles (Fig. 1) reflected the following:

1. Compulsive Non-Avoidant (CNA; *n* = 14): mild-moderate compulsivity, low behavioral avoidance and mild stress (or good perceived ability to cope with life stressors); low CAR; negative learning bias.
2. Compulsive Reactive (CR; *n* = 18): mild-moderate compulsivity, mildly elevated behavioral avoidance and mild stress (or good ability perceived to cope with life stressors); high CAR; strong positive learning bias.
3. Compulsive Stressed (CS; *n* = 13): moderate-severe compulsivity, highly elevated behavioral avoidance and very high stress (or poor perceived ability to cope with life stressors); moderate CAR; positive learning bias.

Subtype differences were assessed on demographic and input variables (Table 1), as well as on validators (i.e., IUC, urgency, anxiety, and depression; Table 1 and Fig. 1). Results of MANOVAs, ANOVAs, and Chi-squared tests are detailed in Supplementary Results, Table S5 and Figs. S3, S4, and S5.

**Table 1:** Demographic and subtype profiles for main input and validating variables.

Subtype	1 (*n* = 14)	2 (*n* = 18)	3 (*n* = 13)	Post hoc comparisons (*p* < 0.05)	Effect size (η_*p*_^2^)
	CNA	CR	CS		
	*M (SD)*	*M (SD)*	*M (SD)*		
Age	24.57 (4.86)	24.56 (4.90)	26.31 (7.77)	*p* = 0.52	
Sex (m/f)	7/7	11/7	2/11	*p* = 0.036	
Primary compulsion (Add/OC)	8/6	6/12	4/9	*p* = 0.67	
FD	0.11 (0.053)	0.11 (0.065)	0.085 (0.028)	*p* = 0.47	
**Measures used in cluster formation**					
Y-BOCS Total	15.57 (5.60)	13.22 (4.62)	23.00 (4.20)	1 < 3; 2 < 3	0.38
Behavioral Avoidance (MEAQ-BA)	30.36 (6.69)	37.78 (6.67)	46.77 (9.44)	1 < 2; 1 < 3; 2 < 3	0.42
Coping with stress (PSS)	18.14 (2.80)	19.28 (3.29)	27.00 (3.79)	1 < 3; 2 < 3	0.55
CAR salience	0.027 (0.22)	0.394 (0.18)	0.210 (0.19)	1 < 2	0.39
Valence learning bias	−0.093 (0.15)	0.213 (0.23)	0.100 (0.20)	1 < 2; 1 < 3	0.30
**Validation measures**					
Anxiety (STAI-Y2)	40.29 (6.07)	42.83 (6.65)	49.69 (6.40)	1 < 3; 2 < 3	0.25
Depression (CESD-R)	8.93 (6.93)	7.22 (4.61)	23.23 (12.04)	1 < 3; 2 < 3	0.39
Intolerance of uncertainty (IUS)	25.00 (5.38)	32.28 (6.28)	40.31 (9.87)	1 < 2; 1 < 3; 2 < 3	0.51
Positive urgency (UPPS-P)	23.21 (6.87)	31.50 (6.00)	33.08 (6.21)	1 < 2; 1 < 3	0.32
Negative urgency (UPPS-P)	24.57 (6.21)	25.94 (3.84)	31.62 (3.89)	1 < 3; 2 < 3	0.25

*Note:* *CNA* Compulsive Non-Avoidant, *CR* Compulsive Reactive, *CS* Compulsive Stressed, *Add.* addiction-related (eating and alcohol) compulsivity, *OC* obsessive compulsive, *FD* Framewise displacement, *Y-BOCS* Yale-Brown Obsessive-Compulsive Scale, *MEAQ-BA* Multidimensional Experiential Avoidance Questionnaire Behavioral Avoidance subscale, *PSS* Perceived Stress Scale, *CAR salience* cortisol awakening response salience score, measured in nanomoles per liter(nmol/L), *STAI-Y2* State-Trait Anxiety Inventory Y2, *CESD* Centre for Epidemiologic Studies Depression Scale Revised, *IUS* Intolerance of Uncertainty Scale, UPPS UPPS-P Impulsive Behavior Scale, *η*_*p*_^*2*^ = partial eta squared.

### Differences in amygdala-based rs-FC between subtypes

**Fig. 2:**
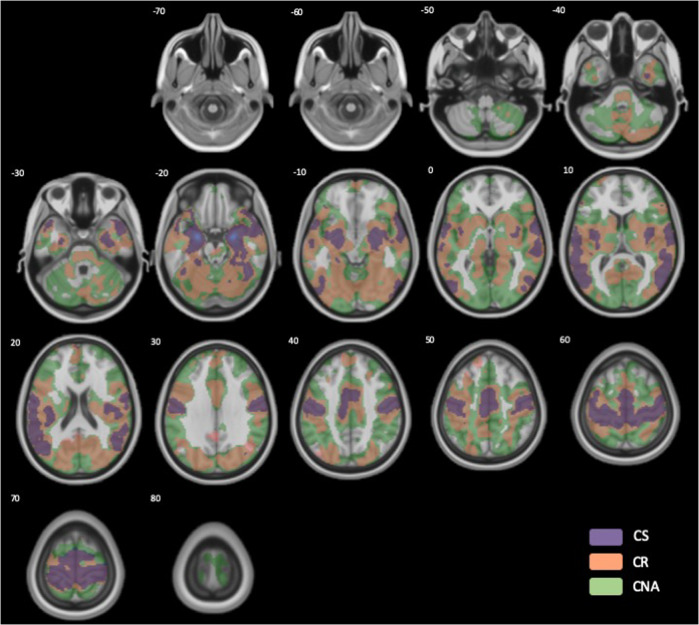
Whole-brain resting-state functional connectivity map for bilateral amygdala seed for three subtypes (threshold used T = 7.7, p = 1e−09). Colors represent brain regions showing functional correlation with amygdala function at rest. CNA Compulsive Non-Avoidant (green), CR Compulsive Reactive (orange), CS Compulsive Stressed subtype (purple).

Subtypes showed no differences in framewise displacement (Table 1), indicating rs-FC findings were not due to motion artifact. Whole-brain analysis of amygdala-based rs-FC revealed connectivity patterns largely consistent with previous studies [76, 77] and showed functional coupling between the amygdala and regions within affect processing networks [55]. Figure 2 illustrates the whole-brain resting-state functional connectivity map for bilateral amygdala seed for the three subtypes at the same threshold (*T* = 7.7, *p* = 1e^−09^). The CNA subtype demonstrated the greatest, widespread functional synchronicity between the amygdala and other brain regions, while the CS group exhibited the least brain regions functionally synchronized with the amygdala. The CR subtype demonstrated a functional connectivity pattern more widespread than the CS subtype, albeit more constrained than the CNA subtype.

**Fig. 3:**
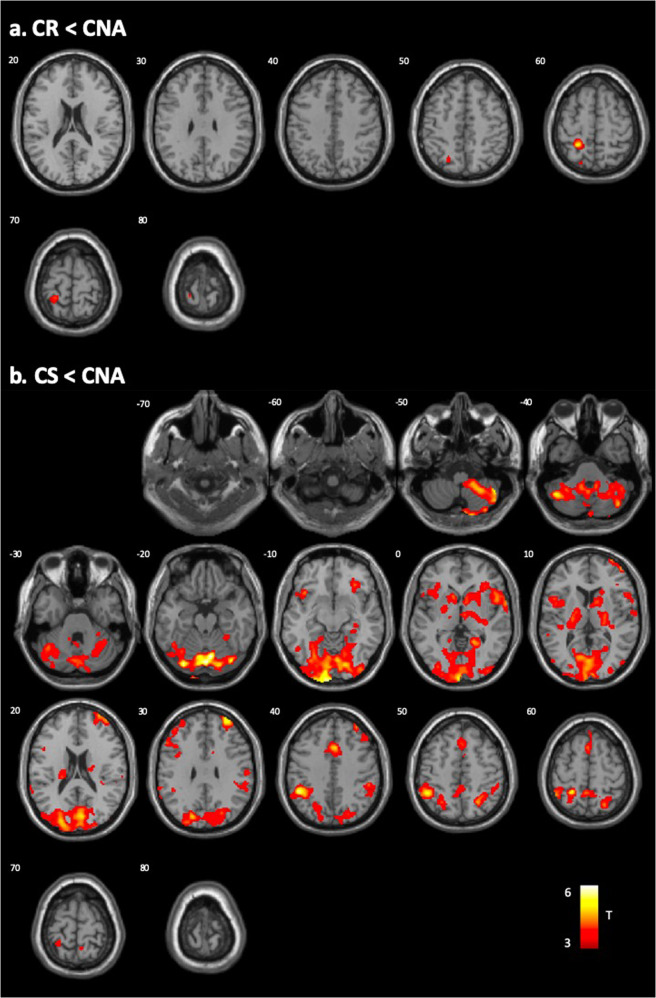
Brain regions showing reduced amygdala-based resting-state functional connectivity between subtypes. **a** CR Compulsive Reactive subtype compared to the CNA Compulsive Non-Avoidant subtype, and **b** CS Compulsive Stressed subtype compared to the CNA subtype. Colored areas indicate significant regions after family-wise correction at the cluster level (*P*_FWE_ < 0.05).

Further statistical group comparisons revealed the CR subtype exhibited significantly decreased functional connectivity of the amygdala at the left superior parietal lobe when compared to the CNA subtype (Table 2; Fig. 3a). The CS subtype demonstrated decreased amygdala functional connectivity at several regions compared to the CNA subtype (Fig. 3b). These included multiple regions within the frontal and temporal lobes, the insula, cerebellum, cuneus, precuneus, superior parietal lobe, and middle occipital gyrus, as well as subcortical regions, including the thalamus, putamen, pallidum, caudate, and nucleus accumbens. No significant differences were observed between the CR and CS subtypes.

**Table 2:** Brain regions exhibiting a significant difference between subtypes in the resting-state functional connectivity of the bilateral amygdala (*p* < 0.001).

*P*_FWE_	*K*	Peak t	MNI coordinates			Hem.	Region
			*x*	*y*	*z*		
**CNA** > **CR**							
0.018	545	5.1	−24	−44	60	L	Superior parietal lobe
**CNA** > **CS**							
<0.001	22,189	6.18	1	−78	−18	R	Cerebellum
		5.74	−30	−80	−22	L	Cerebellum
		4.81	9	−97	4	R	Cuneus
		5.52	−7	−102	−6	L	Cuneus
		4.09	25	−81	−13	R	Middle occipital gyrus
		4.88	−27	−80	−15	L	Middle occipital gyrus
<0.001	2,857	4.71	24	−60	52	R	Precuneus
		4.25	−3	−46	55	L	Precuneus
		4.67	25	−62	53	R	Superior parietal lobe
		4.04	−25	−62	53	L	Superior parietal lobe
		4.37	−4	−46	58	L	Paracentral lobule
		4.46	22	−24	8	R	Thalamus
		3.79	26	−10	7	R	Putamen
		3.54	23	−10	2	R	Pallidum
<0.001	2,203	4.57	52	18	4	R	Inferior frontal gyrus
		4.46	62	6	−2	R	Superior temporal gyrus
		4.00	34	2	−1	R	Insula
		4.41	10	12	4	R	Caudate
0.001	1,080	4.35	−20	−4	6	L	Pallidum
		4.27	−10	14	−2	L	Caudate
		3.79	−15	−22	15	L	Thalamus
		3.71	−25	−1	−3	L	Putamen
		3.91	−12	12	−7	L	Nucleus accumbens
0.001	1,014	4.81	2	18	38	R	Middle cingulate gyrus
		3.90	0	18	56	Mid	Superior motor area
		4.05	−2	21	54	L	Superior frontal gyrus
0.002	929	5.47	32	54	32	R	Superior frontal gyrus
		4.77	34	64	14	R	Middle frontal gyrus
0.006	709	4.51	−38	20	−8	L	Inferior frontal gyrus
		4.19	−50	12	8	L	Precentral gyrus
		3.93	−38	16	−8	L	Insular
0.034	452	4.21	−40	44	32	L	Middle frontal gyrus

*Note:* *MNI* Montreal Neurological Institute, *P*_FWE_ *p*-value after family-wise error correction, *K* cluster size, *Hem.* Hemisphere, *L* Left hemisphere, *R* Right hemisphere, *Mid.* Midline. CNA Compulsive Non-Avoidant, *CR* Compulsive Reactive, *CS* Compulsive Stressed subtype.

Results of supplementary regression analysis to explore independent predictive relationships between key variables and amygdala-based rs-FC are presented in Supplementary Results and Figs. S6 and S7.

## Discussion

A multimodal, data-driven statistical modeling approach was used to identify novel, homogeneous subtypes of transdiagnostic compulsive behavior. Comprising a range of traditional labels (i.e., cleaning, checking, symmetry, compulsive eating, and alcohol use), subtypes identified were independent of behavioral domains and were instead based on the current understanding of shared intermediate affective processes underpinning compulsivity. Each subtype included all types of behavior demonstrating transdiagnostic expression and exhibited unique profiles across psychological, cognitive, and neurobiological indicators. Meaningful differences were observed on validating measures of mood (depression and anxiety) and compulsivity-related constructs (IUC and urgency). IUC and urgency differed systematically in severity across all three subgroups, while mood was only elevated in one subgroup, suggesting the groups were subdivided based on variance in compulsivity and not mood alone. Most importantly, subtypes mapped onto amygdala-based brain network connectivity, illustrating their ability to capture neurobiological distinctiveness and highlighting the relevance of emotion-related brain networks in compulsive behavior.

An important feature of our approach, and other investigations reclassifying mental disorders [17, 19, 20], was the integration of multidimensional indicators to form intermediate phenotypes. This approach can reveal “hidden” subtypes, which demonstrate unique profiles of impairment on indicator variables. Consistent with similar studies in affective and psychotic disorders, multiple subtypes emerged (i.e., CNA, CR, and CS) that exhibited different combinations of impairment on measures of compulsive-emotionality. Subtypes with poorer outcomes exhibited greater reductions on amygdala-based rs-FC.

Subtype CS was most impaired, characterized by moderate-severe compulsivity, over-use of maladaptive emotion regulation strategies (i.e., avoidance), and poor perceived ability to manage stress. Widespread reductions in functional connectivity between the amygdala and nodes within the visual attention network, salience network, DMN, and limbic network were also evident, as was decreased connectivity between the amygdala and cerebellum. The cerebellum is intrinsically connected to the amygdala [78] and is considered a reliable biomarker of emotional states [79] and affective processing [80]. Subtype CNA exhibited mild-moderate levels of compulsive behavior and relatively low/neutral levels across all other indicators, suggesting no obvious emotional processing disruptions. Neurobiologically, there was no evidence of functional connectivity reductions in amygdala linked networks. Subtype CR also demonstrated mild-moderate levels of compulsive behavior, however demonstrated evidence of emotion processing disruptions on other indicators. Subtype CR was characterized by an attentional bias for rewarding stimuli, elevated CAR, and mildly elevated tendency to avoid negative emotions. Reductions in amygdala rs-FC were observed, albeit less pronounced and more localized compared to subtype CS. Reductions were primarily in regions encompassing main nodes of the visual attention and DMN.

The initial classification of compulsivity (i.e., Y-BOCS compulsive subscale score ≥ 5) seems to produce a robust amygdala linked brain network, within which there is further phenotypic variance. There was remarkable consistency between amygdala-based FC reductions and the degree of subtype impairment (Fig. 2). This emphasizes the importance of the amygdala and its network connectivity in explaining individual variance in compulsive behavior. Widespread decreases in rs-FC between limbic regions (amygdala, hippocampus) and other brain networks including basal ganglia, default mode, and attention networks have been found in OCD [57], anxiety, and depression [56, 81]. Decreased functional coupling between the amygdala and cortical/subcortical regions may represent a neural mechanism for increased vulnerability for emotion-driven psychopathology [82–84].

Aspects of the subtype profiles are consistent with past literature and, taken as a whole, reveal processes that may lead to compulsive behavior. The most severe symptom presentation in subtype CS is consistent with previous findings linking elevated stress to increased pathological repetitive behavior in addictions [43, 85, 86] and OCD [87]. Stress promotes habitual behavior [88] and stress hormones (e.g. cortisol) have been argued to reduce goal-directed control over behavior while increasing connectivity between the amygdala and dorsal striatum (a region implicated in habit learning and action initiation [89–91]). The co-occurrence of stress and elevated symptom severity in subtype CS could reflect the ability of stress to turn trait-driven behavioral tendencies into habitual, compulsive behaviors.

Despite reporting the greatest level of stress, subtype CS exhibited only a moderately elevated CAR relative to other subtypes. The relationship between stress and the CAR may present in an inverted-U shaped manner, whereby the CAR is greater under conditions where people actively cope with stressors, while in more severely stressful conditions where coping is reduced, a decrease in the CAR starts to occur [40, 92, 93]. This likely reflects cortisol levels increasing with symptom associations until a threshold is reached and the HPA-axis is down-regulated [94].

By comparison, subtype CR exhibited an elevated CAR coupled with low self-reported stress. The combination of an elevated CAR and low self-reported distress response to stress could be seen as reflecting the link between increased CAR and biological preparedness to actively manage stressors [95]. CR subtype was further differentiated by a strong propensity towards visual reward learning. Reward learning biases on the same task have been linked to increased impulsivity [96], a construct thought to overlap and increase the risk for compulsivity [1]. This finding was validated on self-report measures, which showed this subtype experienced elevated urgency toward positive stimuli/emotions. Increased reward learning, coupled with behavioral avoidance tendencies (i.e., use of behaviors to avoid uncomfortable emotions) and a biological stress-related undertone, may interact to increase vulnerability (albeit mildly) to compulsive behavior. This interpretation is supported neurobiologically by amygdala functional connectivity disruptions between regions within the visual attention and DMN, responsible for visual perception of stimuli which elicit emotional responses and appraisal of emotional stimuli [55].

Subtype CNA appeared most analogous to a healthy group. They demonstrated low self-reported stress and avoidance behaviors and a weak punishment learning bias on the learning task, a finding common within the general population [49]. The low CAR coupled with low stress, suggests minimal daily life stressors. Given the absence of functional disruptions on amygdala-based brain imaging, emotion processing disruptions may not contribute to compulsive behavior in this subtype. Behavior may be better explained by contributory factors not examined here or represent normal human function.

The clinical utility of subtypes ultimately rests on their ability to inspire new research avenues and guide precise treatment recommendations. Treatments for subtype CS could focus on developing adaptive emotion regulation strategies and improving tolerance for negative emotions. Improvements may be visible on amygdala resting-state endpoints. In light of the CR profile, cognitive recalibration of reward/approach attentional biases [97] offers a therapeutic avenue. This subtype presents a target for preventative interventions and investigating risk predictions. Given the elevated CAR and emerging avoidance tendencies, they may be at risk for progression of pathological behavior. This is further supported by emerging disruptions in amygdala network connectivity. Finally, subtype CNA encourages examination of alternative models for classifying compulsive behavior, including reward-based models involving the ventral striatum and related neural networks [25, 98].

This proof-of-concept study represents the first of its kind in the area of compulsivity. Results demonstrate the promise of this approach in generating new understandings of compulsive behavior. Although there are limitations associated with clustering methods [99], precautions were taken to assess the validity of subtypes. Meaningful differences on amygdala rs-FC indicate subtypes were valid representations of underlying neurobiological variance. Although relatively small sample sizes are acceptable in clustering [100], the current study sample was particularly small. This limited the statistical validation techniques that could be used and makes it difficult to confirm the stability of the clusters. Future studies with larger sample sizes may complement our approach with other validation techniques (e.g., split sample and out-of-sample replication) or run alternative clustering methods [101]. A larger sample size may also allow for additional clusters/subtypes in the data to be uncovered [102].

For convenience, and in line with previous studies [65], compulsivity was quantified using total Y-BOCS scores across disorders, which incorporates obsessions and compulsions (both of which are highly correlated and intrinsically linked [66, 103]). Nonetheless, future work could consider other conceptualizations of compulsivity. Our analyses utilized a general community sample with mild to moderate levels of compulsive behavior and thus did not capture healthy controls nor more severe clinical presentations. Pre-selecting vulnerable individuals with at least mild symptoms makes it difficult to draw conclusions about the nature of the subtype profiles in a healthy sample. Similarly, subtype profiles and brain network connectivity disruptions may manifest differently in clinical samples. Longitudinal investigations, additionally incorporating healthy control participants (i.e., those with low/normal levels of compulsive tendencies), could clarify how subtypes and their neural substrates evolve over time, from few to mild/moderate manifestations to severe compulsive behavior.

Finally, this study concerned the likely role of poor affect regulation capacity in compulsive behavior and thus focused on affect-related neurobiological processes. Future research should seek to replicate and extend this approach using “cold” cognitive processes and their neurobiological correlates (e.g. impulsivity, cortical-striatal-thalamic-cortical loops).

## Supplementary information

Supplementary Material
