# Supplementary material for: Transdiagnostic phenotypes of compulsive behavior and associations with psychological, cognitive, and neurobiological affective processing
Source: Transl Psychiatry. 2022 Jan 10;12:10. doi: 10.1038/s41398-021-01773-1 (PMC8748429; doi:10.1038/s41398-021-01773-1)
Supplement: Supplementary file 1 — Supplementary Material [file 41398_2021_1773_MOESM1_ESM.docx]

**SUPPLEMENTARY METHODS AND MATERIALS**

**Participants**

Participants were recruited from the general community. This was done through flyers and online advertisement, primarily targeted at the Monash University Clayton and Caulfield campuses and surrounding areas. As majority of individuals with OCD- and addiction-related behaviors are not in clinical care, this was considered an appropriately representative area. These samples are also considered diverse with respect to race/ethnicity and sex, though generally younger with respect to age.

Participants had no lifetime history of DSM-5 defined psychotic illness, bipolar affective disorder (I and II), bulimia nervosa or anorexia, severe substance use disorder, learning difficulty, ADHD or other condition involving cognitive impairment as the primary feature. Current thoughts of suicide or self-harm, as well as severe anxiety or depression were excluded. There was no history of neurological illness or brain injury, major medical conditions, endocrine disorder, adrenal dysfunction, autoimmune disorder, or other conditions known to have a direct effect on the HPA-axis. Participants were also excluded if currently using psychoactive medications (i.e. antidepressants, mood stabilizers, antipsychotics, benzodiazepines, or other psychiatric medications) or glucocorticoid medications.

**Materials**

**Screening measures.** Screening for inclusion/exclusion criteria involved the MINI International Neuropsychiatric Interview for DSM-5 (M.I.N.I; ^1^, the 7-item Generalized Anxiety Disorder (GAD-7; ^2^, 9-item Patient Health Questionnaire (PHQ-9; ^3^ and Y-BOCS compulsivity subscale ^4^.

**Compulsivity.** Participants were first presented a thresholding question to determine current engagement in a behavior, evident over the past three months. For more occasional behaviors (i.e. gambling and drinking alcohol), participants were asked “*Have you gambled in the past 3 months?”.* For everyday behaviors (i.e. checking, symmetry, cleaning, alcohol consumption and binge-eating), the thresholding question asked about excessive engagement in the behavior. For example, *“In the past 3 months, have you needed to eat food excessively, even though you were not hungry?”.* If participants endorsed a behavior at thresholding, they were then shown an adapted version of the Y-BOCS. The Y-BOCS is a 10-item self-report scale. The first 5 items ask about thoughts related to a behavior (e.g. “*How much of your time was occupied by thoughts of…*”) and second five items are related to the behavior itself (e.g. “*How much time did you spend on…*.”). Individual items of the Y-BOCS were tailored to each behavioral domain being measured. For example, “*How anxious or distressed do you feel if prevented from eating?”* or *“How anxious or distressed do you feel if prevented from drinking alcohol?”.* Participants responded on 5-point Likert scale ranging from 0 - 4 and scores can range from 0 to 40. Higher scores are indicative of greater of severity obsessions and compulsions related to a behavior.

**Behavioral Avoidance.** The behavioral avoidance subscale is made up of 11-items. Participants respond on a 6-point Likert scale ranging from 1(strongly disagree) to 6 (strongly agree). Scores can range from 11 to 66. Higher scores are indicative of increased use of behavioral avoidance strategies to avoid uncomfortable experiences (e.g. “*I work hard to avoid situations that might bring up unpleasant thoughts and feelings in me”*).

**Stress.** It is a 10-item scale, to which participants respond 0 (never) to 4 (very often). Scores range from 0-49 and higher scores indicate greater perceived stress (e.g. “*how often have you been upset because of something that happened unexpectedly”)*.

**Self-reported symptoms: used for validation of subtypes.** Anxiety and depressive symptoms were measured using the State-Trait Anxiety Inventory Y2 (STAI-Y2; ^5^ and Centre for Epidemiologic Studies Depression Scale Revised ^6^, respectively. Constructs which have previously been linked to transdiagnostic compulsivity were also assessed, including intolerance of uncertainty and impulsivity ^7^. This was done using the Intolerance of Uncertainty Scale (IUS; ^8^ and the negative and positive urgency subscales of the UPPS-P Impulsive Behavior Scale ^9^.

**Neurocognitive measure: Full description of cognitive task (BeanFest).** Participants begin the game with 50 points and aim to win by reaching 100 points, and avoid losing by reaching 0 points. Approaching positive beans adds points (+10), while approaching negative beans loses points (-10). Avoiding a bean results in no net loss/gain. Participants receive feedback about the beans value irrespective of whether they decide to approach or avoid it. The beans vary systematically in terms of shapes (i.e. circular to oblong) and how many speckles they have. In the learning phase, participants complete three 36-trial blocks to learn the valence of 36 “game” beans. In the test phase, participants randomly view the 36 game beans, as well as 64 “novel” beans and are asked to indicate if that bean is “helpful” or “harmful”. For further task information, see ^10^.

**Neurohormonal measure: Saliva sampling protocol and analysis.** Participants were asked to collect six saliva samples at home on two consecutive typical working days (awakening (t_0_), 30-minutes after awakening (t_30_) and 45-minutes after awakening (t_45_)). During saliva collection period, they were instructed to take nil by mouth other than water, and not to smoke or brush their teeth. Samples were placed in participants’ home freezer as soon as possible after collection of saliva and transferred to the laboratory in insulated cold packs to be stored at - 20C until assay. Participants were asked to fill in a record sheet on each day recording awakening time and time of collection of saliva samples. Responses were screened for inconsistencies between awakening time and time of first saliva collection on each day. No participants reported a discrepancy of greater than 5 minutes.

Samples were thawed and centrifuged at 1500rpm x g for 15 minutes. Cortisol concentration was determined by salivary cortisol immunoassay kit developed by Salimetrics LLC (USA). Sensitivity = 0.003 μg/dL. Salivary cortisol correlated well with matched serum cortisol concentrations (*r* = 0.91). Intra and inter-assay variations were both below 5%.

To address the problem of non-adherence to the requested saliva sampling regime, suspected non-adherence was examined by identifying CAR profiles showing no cortisol rise from waking sample to either the 30 or 45 min samples post awakening ^11^. Such cases were identified as missing data. As CAR was measured on two consecutive days, when there was a missing data point for one day, the alternative day was used to determine the participants CAR. Where two days were available, an average value of both days was used.

**Detailed image pre-processing.** Dicom images were firstly converted to nifty (i.e., analyse format) using dcm2niix, and organized in BIDS format (https://bids.neuroimaging.io/). The following processed were conducted by fMRIPrep [version 1.1.1 ^12,13^] on a CENTOS 7 cluster computing system ([www.massive.org.au](http://www.massive.org.au)). And the details below were adapted from the method session of fMRIPrep report.

*T1-weighted image:* The T1-weighted (T1w) image was corrected for intensity non-uniformity (INU) with N4BiasFieldCorrection ^14^, distributed with ANTs 2.2.0 ^15^, and used as T1w-reference throughout the workflow. The T1w-reference was then skull-stripped with a Nipype implementation ^16,17^ of the antsBrainExtraction.sh workflow (from ANTs), using OASIS30ANTs as target template. Brain tissue segmentation of cerebrospinal fluid (CSF), white-matter (WM) and gray-matter (GM) was performed on the brain-extracted T1w using fast [FSL 5.0.9, RRID:SCR_002823, ^18^]. Volume-based spatial normalization to standard space (MNI152NLin2009cAsym) was performed through nonlinear registration with antsRegistration (ANTs 2.2.0), using brain-extracted versions of both T1w reference and the T1w template.

*Resting state functional MRI:* First, a reference volume and its skull-stripped version were generated using a custom methodology of fMRIPrep. A deformation field to correct for susceptibility distortions was estimated based on fMRIPrep 2019s fieldmap-less approach. The deformation field is that resulting from co-registering the BOLD reference to the same-subject T1w-reference with its intensity inverted ^19,20^. Registration is performed with antsRegistration (ANTs 2.2.0), and the process regularized by constraining deformation to be nonzero only along the phase-encoding direction, and modulated with an average fieldmap template ^21^. Based on the estimated susceptibility distortion, an unwarped BOLD reference was calculated for a more accurate co-registration with the anatomical reference. The BOLD reference was then co-registered to the T1w reference using flirt [FSL 5.0.9, ^22^]. Co-registration was configured with nine degrees of freedom to account for distortions remaining in the BOLD reference. Head-motion parameters with respect to the BOLD reference (transformation matrices, and six corresponding rotation and translation parameters) are estimated before any spatiotemporal filtering using mcflirt (FSL 5.0.9 ^22^. BOLD images were slice-time corrected using 3dTshift from AFNI 20160207 ^23^. The BOLD time-series were resampled onto their original, native space by applying a single, composite transform to correct for head-motion and susceptibility distortions. These resampled BOLD time-series will be referred to as preprocessed BOLD in original space, or just preprocessed BOLD.

The BOLD time-series were resampled into standard spaces, correspondingly generating the following spatially-normalized, preprocessed BOLD images: MNI152NLin2009cAsym. A reference volume and its skull-stripped version were generated using a custom methodology of fMRIPrep. Automatic removal of motion artifacts using independent component analysis (ICA-AROMA), ^24^ was performed on the preprocessed BOLD on MNI space time-series after removal of non-steady state volumes and spatial smoothing with an isotropic, Gaussian kernel of 6mm FWHM (full-width half-maximum). Corresponding non-aggressively denoised images were produced after such smoothing.

Several confounding time-series were calculated based on the preprocessed BOLD: framewise displacement (FD), DVARS and three region-wise global signals. FD and DVARS are calculated for each functional run, both using their implementations in Nipype (following the definitions by ^25^. The three global signals are extracted within the CSF, the WM, and the whole-brain masks. Additionally, a set of physiological regressors were extracted to allow for component-based noise correction [CompCor, ^26^]. Principal components are estimated after high-pass filtering the preprocessed BOLD time-series (using a discrete cosine filter with 128s cut-off) for the two CompCor variants: temporal (tCompCor) and anatomical (aCompCor). tCompCor components are then calculated from the top 5% variable voxels within a mask covering the subcortical regions. This subcortical mask is obtained by heavily eroding the brain mask, which ensures it does not include cortical GM regions. For aCompCor, components are calculated within the intersection of the aforementioned mask and the union of CSF and WM masks calculated in T1w space, after their projection to the native space of each functional run (using the inverse BOLD-to-T1w transformation). Components are also calculated separately within the WM and CSF masks. For each CompCor decomposition, the k components with the largest singular values are retained, such that the retained components\u2019 time series are sufficient to explain 50 percent of variance across the nuisance mask (CSF, WM, combined, or temporal). The remaining components are dropped from consideration. The head-motion estimates calculated in the correction step were also placed within the corresponding confounds file. The confound time series derived from head motion estimates and global signals were expanded with the inclusion of temporal derivatives and quadratic terms for each (Satterthwaite et al. 2013). Frames that exceeded a threshold of 0.5 mm FD or 1.5 standardized DVARS were annotated as motion outliers. All resamplings can be performed with a single interpolation step by composing all the pertinent transformations (i.e. head-motion transform matrices, susceptibility distortion correction when available, and co-registrations to anatomical and output spaces). Gridded (volumetric) resamplings were performed using antsApplyTransforms (ANTs), configured with Lanczos interpolation to minimize the smoothing effects of other kernels ^27^. Non-gridded (surface) resamplings were performed using mri_vol2surf (FreeSurfer).

*Detailed image post-processing:* SPM12 (matlab r2018) was used to conduct the voxel wise statistical analysis. Firstly, all the output images of amygdala-based rs-FC maps from preprocessing were used to generate the rs-FC patterns for each group using three separate one-sample t-tests. For illustration purposes, a stringent threshold (T = 7.7, p = 1e-09) was applied for three subgroups. Secondly, to further statistically explore the group differences, the F-test mode is used (F-test with controlling covariance on SPM12), with group as main factor (3 levels) and controlling for age and sex. F-contrast was setup to detect any group differences among three subgroups. Post-hoc independent t-tests were conducted to examine directional differences between each of the subgroups. For each comparison, results were thresholded at uncorrected p-value < .001 with cluster size > 10, then corrected for multiple comparisons error at the cluster level of p < .05, using family wise error (FWE) correction. Only p__FWE_<0.05 regions were considered as significant.

**SUPPLEMENTARY RESULTS**

**Missing data, outliers and normality**

Missing values create problems for clustering approaches and omitting entire cases with one missing domain decreases the sample size considerably. Therefore, before performing clustering, any missing values were approximated. Most participants accurately completed all measures, however some participants had missing data for the CAR due to saliva samples not being returned (*n* = 4, 8.89%) or suspected non-adherence to the requested saliva sampling regime (*n* = 2, 4.44%). This data was considered missing at random and approximated using Expectation Maximisation procedures ^28^. There were no other missing data. There was one univariate outlier in the data for the valence learning bias (*z* = 3.32), which was dealt with using winsorising ^29^. Multivariate outliers were not identified on the study sample with the critical value of Mahalanobis distance χ^2^(5) > 20.51, *p* < .001. Skewness and kurtosis were also examined for all variables to be entered into the cluster analysis. Skewness and kurtosis values were converted to *z* values, which ranged from – 0.40 to 1.46 and – 0.74 and 0.07 respectively. These values did not fall outside the critical value *z* = ± 2.58, *p* < .01, indicating no deviations from normality ^28^. There was also no evidence of multicollinearity between variables.

Table S1.

Descriptive characteristics of measures entered into cluster analysis

| Measure | Mean (*SD*); Range |
| --- | --- |
| Y-BOCS | 16.76 (6.31); 5 – 31 |
| MEAQ-BA | 38.07 (9.80); 20 - 63 |
| PSS | 21.16 (4.98); 12 - 34 |
| CAR salience (nmol/L) | .23 (.25); - .26 - .65 |
| Valence learning bias | .09 (.24); - .38 - .60 |

*Note:* Y-BOCS = Yale-Brown Obsessive-Compulsive Scale; MEAQ-BA = Multidimensional Experiential Avoidance Questionnaire Behavioural Avoidance subscale; PSS = Perceived Stress Scale; CAR salience = cortisol awakening response salience score, measured in nanomoles per litre (nmol/L).

Table S2.

Pearson correlations for measures entered into the cluster analysis.

|  | Y-BOCS | MEAQ-BA | PSS | CAR salience (nmol/L) | Valence learning bias |
| --- | --- | --- | --- | --- | --- |
| Y-BOCS |  |  |  |  |  |
| MEAQ-BA | .33* |  |  |  |  |
| PSS | .52** | .48** |  |  |  |
| CAR salience (nmol/L) | - .11 | .20 | - .06 |  |  |
| Valence learning bias | - .19 | .05 | .02 | .13 |  |

** Correlation is significant at the .01 level.

* Correlation is significant at the .05 level.

Table S3.

Agglomeration schedule from hierarchical cluster analysis

| Stage | Cluster Combined | | Coefficients | Stage Cluster First Appears | | Next Stage |
| --- | --- | --- | --- | --- | --- | --- |
|  | Cluster 1 | Cluster 2 |  | Cluster 1 | Cluster 2 |  |
| 1 | 24 | 25 | 0.112 | 0 | 0 | 8 |
| 2 | 11 | 18 | 0.341 | 0 | 0 | 16 |
| 3 | 15 | 16 | 0.611 | 0 | 0 | 15 |
| 4 | 20 | 35 | 0.991 | 0 | 0 | 16 |
| 5 | 3 | 5 | 1.5 | 0 | 0 | 26 |
| 6 | 27 | 32 | 2.036 | 0 | 0 | 24 |
| 7 | 26 | 40 | 2.592 | 0 | 0 | 18 |
| 8 | 23 | 24 | 3.166 | 0 | 1 | 11 |
| 9 | 42 | 44 | 3.801 | 0 | 0 | 29 |
| 10 | 39 | 41 | 4.452 | 0 | 0 | 32 |
| 11 | 22 | 23 | 5.135 | 0 | 8 | 25 |
| 12 | 28 | 30 | 5.868 | 0 | 0 | 25 |
| 13 | 2 | 4 | 6.672 | 0 | 0 | 19 |
| 14 | 10 | 21 | 7.522 | 0 | 0 | 17 |
| 15 | 13 | 15 | 8.379 | 0 | 3 | 20 |
| 16 | 11 | 20 | 9.471 | 2 | 4 | 31 |
| 17 | 10 | 31 | 10.656 | 14 | 0 | 27 |
| 18 | 26 | 29 | 11.867 | 7 | 0 | 32 |
| 19 | 2 | 7 | 13.102 | 13 | 0 | 30 |
| 20 | 13 | 14 | 14.36 | 15 | 0 | 31 |
| 21 | 33 | 34 | 15.851 | 0 | 0 | 37 |
| 22 | 37 | 38 | 17.347 | 0 | 0 | 35 |
| 23 | 6 | 9 | 18.909 | 0 | 0 | 30 |
| 24 | 27 | 36 | 20.592 | 6 | 0 | 35 |
| 25 | 22 | 28 | 22.301 | 11 | 12 | 28 |
| 26 | 3 | 45 | 24.087 | 5 | 0 | 34 |
| 27 | 10 | 12 | 26.011 | 17 | 0 | 40 |
| 28 | 17 | 22 | 28.821 | 0 | 25 | 33 |
| 29 | 42 | 43 | 31.808 | 9 | 0 | 38 |
| 30 | 2 | 6 | 34.886 | 19 | 23 | 36 |
| 31 | 11 | 13 | 38.38 | 16 | 20 | 37 |
| 32 | 26 | 39 | 41.955 | 18 | 10 | 41 |
| 33 | 17 | 19 | 46.727 | 28 | 0 | 41 |
| 34 | 3 | 8 | 51.78 | 26 | 0 | 39 |
| 35 | 27 | 37 | 56.851 | 24 | 22 | 40 |
| 36 | 1 | 2 | 62.199 | 0 | 30 | 38 |
| 37 | 11 | 33 | 69.939 | 31 | 21 | 42 |
| 38 | 1 | 42 | 77.911 | 36 | 29 | 39 |
| 39 | 1 | 3 | 86.497 | 38 | 34 | 44 |
| 40 | 10 | 27 | 95.338 | 27 | 35 | 42 |
| 41 | 17 | 26 | 110.94 | 33 | 32 | 43 |
| 42 | 10 | 11 | 134.632 | 40 | 37 | 43 |
| 43 | 10 | 17 | 162.337 | 42 | 41 | 44 |
| 44 | 1 | 10 | 220 | 39 | 43 | 0 |

Table S4.

Percentage (%) change in agglomeration coefficient and rationale for cluster selection

| Cluster solution | % Change | Rationale |
| --- | --- | --- |
| 9 | 12.44 |  |
| 8 | 11.40 |  |
| 7 | 11.02 |  |
| 6 | 10.22 |  |
| 5 | 16.36 | % change lower than average |
| 4 | 21.36 | increase larger than previous stage; stopping point |
| 3 | 20.58 | 3 cluster is next favored solution |
| 2 | 35.52 | largest change for 2 cluster solution |
| 1 |  |  |
| Average % change | 17.36 |  |


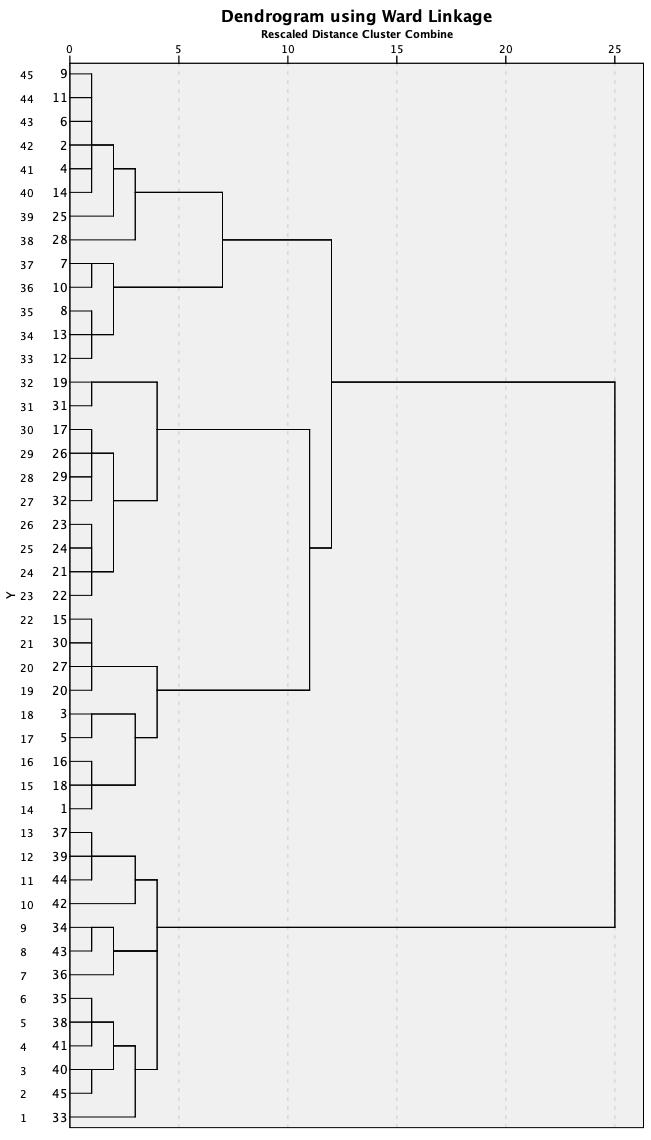


**Fig S1:** Dendrogram for hierarchical agglomerative method (Ward’s method) with squared Euclidean distance. The x-axis represents the degree of dissimilarity between cases, measured via the squared Euclidean distance. The y-axis represents pairs of cases, where numbers refer to the SPSS line rather than subject ID.


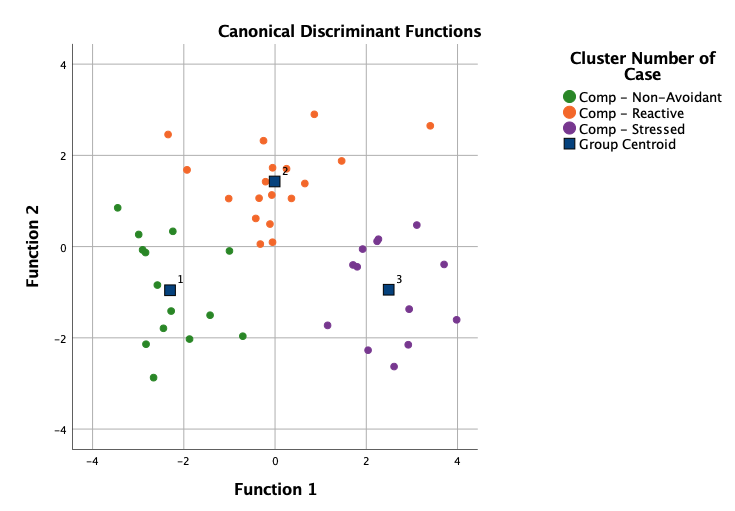


**Fig S2**. Three cluster solution plotted in discriminant function space.

**Validation of final cluster solution: MANOVA’s, ANOVA’s and Chi-square tests**

Subgroups were compared on demographic variables. A chi-squared test for independence indicated there was a significant association between sex and cluster membership, $\chi$^2^ (1, *n* = 45) = 6.65, *p* = .036, *phi* = .38. Sex was therefore included as a covariate in all subsequent analyses. There was no significant association between cluster and compulsion type (i.e. OC-related or behavior-related), $\chi$^2^ (1, *n* = 45) = 1.31, *p* = .52, *phi* = .52, or age, *F*(2, 42) = .41, *p* = .67.

In the assessment of subgroup differences, multivariate general linear models (GLM) with a factor of group (3: cluster 1, 2 or 3) and covariate of sex, yielded significant differences (*p* < .05) in compulsivity (i.e. Y-BOCS total, obsessions and compulsions subscales) and compulsivity-related variables (i.e. IUS, UPPS-P positive and negative urgency subscales), *F* (10, 76) = 8.41, *p* < .001; Pillai’s Trace = 1.06, η_p_^2^ = .53. Tests of between-subjects effects indicated significant differences on the Y-BOCS total (*F*(2, 41) = 12.36, *p* <.001, η_p_^2^ = .38), Y-BOCS obsessions subscale (*F*(2, 41) = 11.55, *p* <.001, η_p_^2^ = .36), Y-BOCS compulsions subscale (*F*(2, 41) = 10.29, *p* <.001, η_p_^2^ = .33), IUS (*F*(2, 41) = 21.65, *p* <.001, η_p_^2^ = .51), UPPS-P negative (*F*(2, 41) = 6.93, *p* = .003, η_p_^2^ = .25) and positive (*F*(2, 41) = 9.77, *p* <.001, η_p_^2^ = .32) urgency subscales. Post-hoc with Bonferroni adjustment for multiple comparisons were used to identify group differences (Table S4). Subgroup 3 exhibited poorer outcomes on most measures of compulsivity and compulsivity-related variables when compared to subgroup 1 and 2. Subgroup 2 demonstrated higher levels of intolerance to uncertainty (IUS) and positive urgency (UPPS-P) in comparison to cluster 1.

Multivariate GLM also demonstrated significant differences on behavioral avoidance (i.e. MEAQ-behavioral avoidance subscale) and psychological wellbeing variables (i.e. PSS, STAI-Y2, CESD-R), *F* (8, 78) = 6.68, *p* < .001; Pillai’s Trace = .81, η_p_^2^ = .41. Tests of between-subjects effects indicated significant differences on the MEAQ-BA (*F*(2, 41) = 14.84, *p* < .001, η_p_^2^ = .42), PSS (*F*(2, 41) = 24.79, *p* < .001, η_p_^2^ = .55), STAI-Y2 (*F*(2, 41) = 6.99, *p* = .002, η_p_^2^ = .25) and CESD-R (*F*(2, 41) = 12.91, *p* <.001, η_p_^2^ = .39). Post-hoc comparisons for group differences are displayed in Table S4. Again, subgroup 3 showed significantly poorer psychological wellbeing than subgroups 1 and 2 across most measures. Subgroup 2 demonstrated significantly higher behavioral avoidance compared to cluster 1.

Univariate GLM with a factor of group (3: cluster 1, 2 or 3) and covariate of sex also yielded significant differences (*p* < .05) in CAR salience score, *F* (2, 41) = 13.14, *p* < .001, η_p_^2^ = .39 and valence learning bias, *F* (2, 41) = 8.97, *p* = .001, η_p_^2^ = .30. Post-hoc comparisons for group differences are displayed in Table S4. Subgroup 2 had a significantly higher CAR when compared to cluster 1. Subgroup 1 had a negative learning bias compared to subgroups 2 and 3, which both demonstrated positive learning biases.

Follow-up investigation using multivariate GLM revealed both the two-cluster (F (5, 36) = 3.64, p = .009; Pillai’s Trace = .34, η_p_^2^ = .34) and the three-cluster (F (10, 74) = 3.64, p < .001; Pillai’s Trace = 1.08, η_p_^2^ = .54) solutions significantly explained the variance in the cluster variables. However, a greater portion of variance was explained by the three-cluster solution (54% variance explained) compared to the two-cluster solution (34% variance explained).

Table S5.

Demographic and cluster profiles

| Subgroup | 1 (*n* = 14) | 2 (*n* = 18) | 3 (*n* = 13) |  |
| --- | --- | --- | --- | --- |
|  | Non-Avoidant | Reactive | Stressed | Post hoc comparisons  (*p* < .05) |
|  | *M (SD)* | *M (SD)* | *M (SD)* |  |
| Age | 24.57 (4.86) | 24.56 (4.90) | 26.31 (7.77) | *p* = .52 |
| Sex (m/f) | 7/7 | 11/7 | 2/7 | *p* = .036 |
| Primary compulsion (Beh/OC) | 8/6 | 6/12 | 4/9 | *p* = .67 |
|  |  |  |  |  |
| *Compulsivity (Y-BOCS)* |  |  |  |  |
| Total | 15.57 (5.60) | 13.22 (4.62) | 23.00 (4.20) | 1 < 3; 2 < 3 |
| Obsessions | 7.57 (2.77) | 6.39 (2.17) | 11.00 (2.34) | 1 < 3; 2 < 3 |
| Compulsions | 8.00 (3.16) | 6.83 (2.81) | 12.00 (2.20) | 1 < 3; 2 < 3 |
|  |  |  |  |  |
| *Psychological wellbeing* |  |  |  |  |
| Behavioral Avoidance (MEAQ-BA) | 30.36 (6.69) | 37.78 (6.67) | 46.77 (9.44) | 1 < 2; 1 < 3; 2 < 3 |
| Coping with stress (PSS) | 18.14 (2.80) | 19.28 (3.29) | 27.00 (3.79) | 1 < 3; 2 < 3 |
| Anxiety (STAI-Y2) | 40.29 (6.07) | 42.83 (6.65) | 49.69 (6.40) | 1 < 3; 2 < 3 |
| Depression (CESD-R) | 8.93 (6.93) | 7.22 (4.61) | 23.23 (12.04) | 1 < 3; 2 < 3 |
|  |  |  |  |  |
| *Compulsivity-related variables* |  |  |  |  |
| Intolerance of uncertainty (IUS) | 25.00 (5.38) | 32.28 (6.28) | 40.31 (9.87) | 1 < 2; 1 < 3; 2 < 3 |
| Positive urgency (UPPS-P) | 23.21 (6.87) | 31.50 (6.00) | 33.08 (6.21) | 1 < 2; 1 < 3 |
| Negative urgency (UPPS-P) | 24.57 (6.21) | 25.94 (3.84) | 31.62 (3.89) | 1 < 3; 2 < 3 |
|  |  |  |  |  |
| *Cortisol Awakening Response* |  |  |  |  |
| CAR salience | .027 (.22) | .394 (.18) | .210 (.19) | 1 < 2 |
| t_0_ | 7.63 (5.02) | 8.20 (4.21) | 9.35 (5.19) | ns |
| MnInc | 6.23 (3.07) | 5.84 (3.31) | 5.01 (5.81) | ns |
|  |  |  |  |  |
| *Cognitive bias (BeanFest)* |  |  |  |  |
| Valence learning bias | - .093 (.15) | .213 (.23) | .100 (.20) | 1 < 2; 1 < 3 |
|  |  |  |  |  |

*Note:* Beh = behavior-related compulsion (i.e. alcohol or eating); OC = obsessive-compulsive related (i.e. checking, symmetry or contamination); Y-BOCS = Yale-Brown Obsessive-Compulsive Scale; MEAQ-BA = Multidimensional Experiential Avoidance Questionnaire Behavioral Avoidance subscale; PSS = Perceived Stress Scale; STAI-Y2 = State-Trait Anxiety Inventory Y2; CESD = Centre for Epidemiologic Studies Depression Scale Revised; IUS = Intolerance of Uncertainty Scale; UPPS = UPPS-P Impulsive Behavior Scale; CAR salience = cortisol awakening response salience score, measured in nanomoles per litre (nmol/L); t_0_ = salivary cortisol on awakening in nmol/L; MnInc = Mean Increase in cortisol from awakening; ns = non-significant difference between groups.

**Fig. S3.** Subgroup differences on psychological cluster variables. MEAQ-BA = Multidimensional Experiential Avoidance Questionnaire Behavioral Avoidance subscale; PSS = Perceived Stress Scale. Bars represent group means and error bars represent standard error. * *p* < .05

**Fig. S4.** Subgroup differences on cortisol awakening response and valence learning bias. Bars represent group means and error bars represent standard error. * *p* < .05

**Fig. S5.** Subgroup differences on compulsivity. Bars represent group means and error bars represent standard error. * *p* < .05

**Follow-up investigations from peer-review.**

Supplementary analyses were conducted as recommended during peer review. A two-group t-test comparing amygdala-based rs-fMRI between participants who did vs did not meet the criteria for DSM diagnosis revealed no significant differences between groups. On self-report validation measures, there were also no significant differences between these groups.

Three regression analyses were also conducted to determine if key variables Compulsivity, Behavioral Avoidance and Stress could predict amygdala rs-fMRI. While we found no effect of Compulsivity, there was a significant negative correlation between Behavioral Avoidance and a related parietal region (including bilateral Cuneus and Calcarine; Figure S6). There were also several regions that negatively correlated with Stress, including Bilateral Caudate, Superior Frontal and Cerebellum (Figure S7). We compared these regions with the regional differences shown between the subgroups in the main manuscript and found that while these key variables likely explained some of the regional differences between subgroups, there still remain unique regions including the precuneus, nucleus accumbens, putamen, thalamus, insula, temporal regions, and frontal regions. This finding implies that although Amygdala FC of some regions are possibly driven by one of the key measurements that determine the subgroups, our subgroups did show unique effects at other brain regions beyond what could be offered by these key measurements alone.


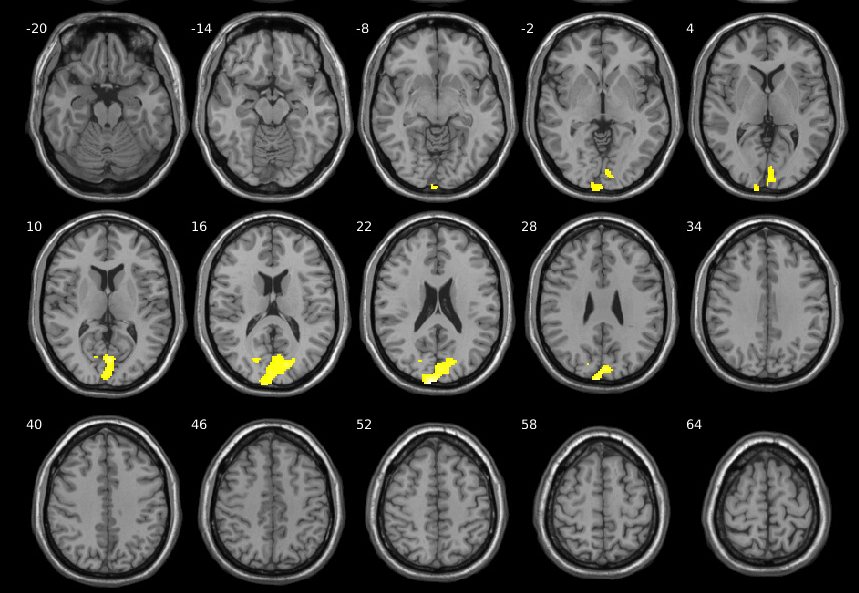


**Fig. S6.** Brain regions (amygdala-based rs-fMRI) that negatively correlated with Behavioral Avoidance (as measured by the MEAQ-BA).


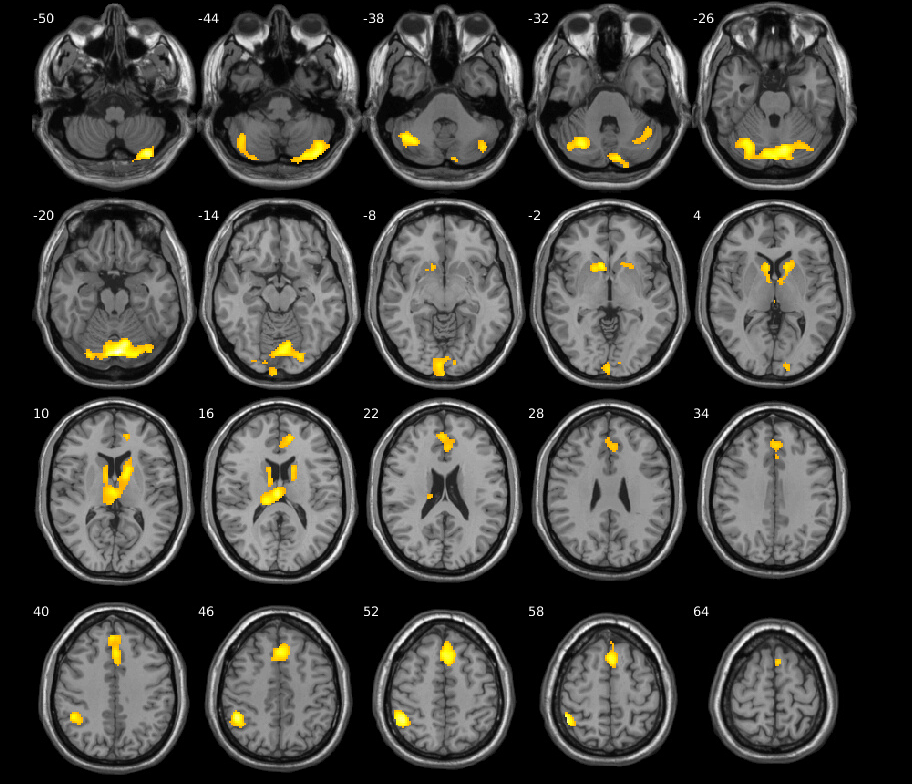


**Fig. S7.** Brain regions (amygdala-based rs-fMRI) that negatively correlated with Stress (as measured by the PSS).

**REFERENCES**

1 Sheehan D V, Lecrubier Y, Sheehan KH, Amorim P, Janavs J, Weiller E *et al.* The Mini-International Neuropsychiatric Interview (M.I.N.I.): the development and validation of a structured diagnostic psychiatric interview for DSM-IV and ICD-10. *J Clin Psychiatry* 1998; **59 Suppl 20**: 22-33;quiz 34-57.

2 Spitzer RL, Kroenke K, Williams JBW, Löwe B. A Brief Measure for Assessing Generalized Anxiety Disorder. *Arch Intern Med* 2006; **166**: 1092.

3 Kroenke K, Spitzer RL, Williams JB. The PHQ-9: validity of a brief depression severity measure. *J Gen Intern Med* 2001; **16**: 606–13.

4 Goodman WK. The Yale-Brown Obsessive Compulsive Scale. *Arch Gen Psychiatry* 1989; **46**: 1006.

5 Spielberger CD, Gorsuch RL, R.E L. *Manual for the State-Trait Anxiety Inventory*. Consulting Psychologists Press: Palo Alto, CA, 1970.

6 Eaton WW, Smith C, Ybarra M, Muntaner C, Tien A. Center for Epidemiologic Studies Depression Scale: Review and Revision (CESD and CESD-R). In: *The use of psychological testing for treatment planning and outcomes assessment: Instruments for adults, Volume 3, 3rd ed.* Lawrence Erlbaum Associates Publishers: Mahwah, NJ, US, 2004, pp 363–377.

7 Tiego J, Oostermeijer S, Prochazkova L, Parkes L, Dawson A, Youssef G *et al.* Overlapping dimensional phenotypes of impulsivity and compulsivity explain co-occurrence of addictive and related behaviors. *CNS Spectr* 2019; **24**: 426–440.

8 Buhr K, Dugas MJ. The Intolerance of Uncertainty Scale: psychometric properties of the English version. *Behav Res Ther* 2002; **40**: 931–45.

9 Lynam DR, Smith GT, Whiteside SP, Cyders MA. The UPPS-P: Assessing five personality pathways to impulsive behavior. West Lafayette, 2006 doi:10.1371/journal.pone.0098996.

10 Fazio RH, Pietri ES, Rocklage MD, Shook NJ. *Positive versus negative valence: Asymmetries in attitude formation and generalization as fundamental individual differences*. 2015 doi:10.1016/bs.aesp.2014.09.002.

11 Thorn L, Hucklebridge F, Evans P, Clow A. Suspected non-adherence and weekend versus week day differences in the awakening cortisol response. *Psychoneuroendocrinology* 2006; **31**: 1009–18.

12 Esteban O, Markiewicz CJ, Blair RW, Moodie CA, Isik AI, Erramuzpe A *et al.* fMRIPrep: a robust preprocessing pipeline for functional MRI. *Nat Methods* 2019; **16**: 111–116.

13 Esteban O, Markiewicz CJ, DuPre E, Goncalves M, Kent JD, Ciric R *et al.* fMRIPrep: a robust preprocessing pipeline for functional MRI. *Zenodo* 2020. doi:10.5281/zenodo.3714205.

14 Tustison NJ, Avants BB, Cook PA, Yuanjie Zheng, Egan A, Yushkevich PA *et al.* N4ITK: Improved N3 Bias Correction. *IEEE Trans Med Imaging* 2010; **29**: 1310–1320.

15 Avants B, Epstein C, Grossman M, Gee J. Symmetric diffeomorphic image registration with cross-correlation: Evaluating automated labeling of elderly and neurodegenerative brain. *Med Image Anal* 2008; **12**: 26–41.

16 Gorgolewski K, Burns CD, Madison C, Clark D, Halchenko YO, Waskom ML *et al.* Nipype: A Flexible, Lightweight and Extensible Neuroimaging Data Processing Framework in Python. *Front Neuroinform* 2011; **5**. doi:10.3389/fninf.2011.00013.

17 Gorgolewski KJ, Esteban O, Ellis DG, Notter MP, Ziegler E, Johnson H *et al.* Nipype: a flexible, lightweight and extensible neuroimaging data processing framework in Python. 0.13.1. 2017. doi:10.5281/ZENODO.581704.

18 Zhang Y, Brady M, Smith S. Segmentation of brain MR images through a hidden Markov random field model and the expectation-maximization algorithm. *IEEE Trans Med Imaging* 2001; **20**: 45–57.

19 Wang S, Peterson DJ, Gatenby JC, Li W, Grabowski TJ, Madhyastha TM. Evaluation of Field Map and Nonlinear Registration Methods for Correction of Susceptibility Artifacts in Diffusion MRI. *Front Neuroinform* 2017; **11**. doi:10.3389/fninf.2017.00017.

20 Huntenburg JM. *Evaluating nonlinear coregistration of BOLD EPI and T1w images. Master Thesis*. 2014.

21 Treiber JM, White NS, Steed TC, Bartsch H, Holland D, Farid N *et al.* Characterization and Correction of Geometric Distortions in 814 Diffusion Weighted Images. *PLoS One* 2016; **11**: e0152472.

22 Jenkinson M, Smith S. A global optimisation method for robust affine registration of brain images. *Med Image Anal* 2001; **5**: 143–156.

23 Cox RW, Hyde JS. Software tools for analysis and visualization of fMRI data. *NMR Biomed* 1997; **10**: 171–178.

24 Pruim RHR, Mennes M, van Rooij D, Llera A, Buitelaar JK, Beckmann CF. ICA-AROMA: A robust ICA-based strategy for removing motion artifacts from fMRI data. *Neuroimage* 2015; **112**: 267–277.

25 Power JD, Mitra A, Laumann TO, Snyder AZ, Schlaggar BL, Petersen SE. Methods to detect, characterize, and remove motion artifact in resting state fMRI. *Neuroimage* 2014; **84**: 320–341.

26 Behzadi Y, Restom K, Liau J, Liu TT. A component based noise correction method (CompCor) for BOLD and perfusion based fMRI. *Neuroimage* 2007; **37**: 90–101.

27 Lanczos C. Evaluation of Noisy Data. *J Soc Ind Appl Math Ser B Numer Anal* 1964; **1**: 76–85.

28 Hair JF, Black WC, Babin BJ, Anderson RE, Tatham RL. *Multivariate data analysis*. Pearson Prentice Hall Upper Saddle River, NJ, 2014.

29 Tabachnick BG, Fidell LS. *Using Multivariate Statistics*. Pearson, 2007https://books.google.com.au/books?id=AkBlQgAACAAJ.
